# Maternal supplementation of different trace mineral sources on broiler breeder production and progeny growth and gut health

**DOI:** 10.3389/fphys.2022.948378

**Published:** 2022-09-23

**Authors:** Fabricia de Arruda Roque, Juxing Chen, Raquel B Araujo, André Luis Murcio, Brunna Garcia de Souza Leite, Mylena Tückmantel Dias Tanaka, Carlos Alexandre Granghelli, Paulo Henrique Pelissari, Rachel Santos Bueno Carvalho, David Torres, Mercedes Vázquez‐Añón, Deana Hancock, Cristiane Soares da Silva Araujo, Lúcio Francelino Araujo

**Affiliations:** ^1^ Universidade de São Paulo, Pirassununga, São Paulo, Brazil; ^2^ Novus International Inc., St. Charles, MO, United States; ^3^ Cobb Vantress, São Paulo, Brazil

**Keywords:** breeder reproduction, bone quality, progeny, intestinal inflammation, egg quality, gut health, chelated trace minerals, maternal supplementation

## Abstract

Trace mineral minerals Zn, Cu, and Mn play important roles in breeder production and progeny performance. The objective of this study was to determine maternal supplementation of trace mineral minerals on breeder production and progeny growth and development. A total of 540 broiler breeders, Cobb 500 (Slow feathering; 0–66 weeks old) were assigned to one of three treatment groups with the same basal diet and three different supplemental trace minerals: ITM–inorganic trace minerals in sulfates: 100, 16, and 100 ppm of Zn, Cu, and Mn respectively; MMHAC -mineral methionine hydroxy analog chelate: 50, 8, and 50 ppm of bis-chelated MINTREX^®^Zn, Cu and Mn (Novus International, Inc.), and TMAAC - trace minerals amino acid complex: 50, 8, and 50 ppm of Zn, Cu, and Mn. At 28 weeks of age, eggs from breeder treatments were hatched for progeny trial, 10 pens with 6 males and 6 female birds per pen were fed a common diet with ITM for 45 days. Breeder production, egg quality, progeny growth performance, mRNA expression of gut health associated genes in breeder and progeny chicks were measured. Data were analyzed by one-way ANOVA; means were separated by Fisher’s protected LSD test. A *p*-Value ≤ 0.05 was considered statistically different and 0.1 was considered numerical trend. Breeders on ITM treatment had higher (*p* < 0.05) body weight (BW), weight gain and lower (*p* < 0.05) feed conversion ratio (FCR) from 0 to 10 weeks, when compared to birds fed MMHAC. MMHAC significantly improved egg mass by 3 g (*p* < 0.05) and FCR by 34 points (0.05 < *p* < 0.1) throughout the reproductive period (26–66 weeks) in comparison to ITM. MMHAC improved (*p* < 0.01) egg yolk color versus (vs.) ITM and TMAAC in all periods, except 28 weeks, increased (*p* < 0.01) eggshell thickness and resistance vs. TMAAC at 58 weeks, and reduced (*p* < 0.05) jejunal NF-κB gene expression vs. TMAAC at 24 weeks. There was a significant reduction in tibial dry matter weight, Seedor index and resistance for the breeders that received MMHAC and/or TMAAC when compared to ITM at 18 weeks. Lower seedor index but numerically wider tibial circumference was seen in hens fed MMHAC at 24 weeks, and wider tibial circumference but lower tibial resistance in hens fed TMAAC at 66 weeks. Maternal supplementation of MMHAC in breeder hens increased (*p* < 0.0001) BW vs. ITM and TMAAC at hatching, reduced (*p* < 0.05) feed intake vs. ITM at d14 and d28, and improved (*p* < 0.01) FCR and performance index vs. TMAAC at d28, reduced (*p* < 0.01) NF-κB gene expression and increased (*p* < 0.05) A20 gene expression vs. TMAAC on d0 and vs. ITM on d14, reduced (*p* < 0.05) TLR2 gene expression vs. ITM on d0 and vs. TMAAC on d14, increased (*p* < 0.05) MUC2 gene expression vs. both ITM and TMAAC on d45 in progeny jejunum. Overall, these results suggest that supplementation with lower levels of MHA-chelated trace minerals improved breeder production and egg quality and reduced breeder jejunal inflammation while maintaining tibial development in comparison to those receiving higher inorganic mineral supplementation, and it also carried over the benefits to progeny with better growth performance, less jejunal inflammation and better innate immune response and gut barrier function in comparison to ITM and/or TMAAC.

## Introduction

Trace minerals such as zinc (Zn), copper (Cu), and manganese (Mn) are essential cofactors for hundreds of cellular enzymes and transcription factors in all animal species, including poultry, and are integral in a wide variety of biochemical and physiological processes. Zn plays a significant role in many biological processes, such as cell proliferation and animal growth, immune system development and response, reproduction, gene regulation, and protection against oxidative stress and damage (Zhao et al., 2016; Qi et al., 2019; Londero et al., 2020). Like Zn, Cu is essential for a wide variety of health and performance-related functions in all animal species. For instance, Cu regulates collagen crosslinking therefore promoting skin, bone, tendon and intestinal integrity (Rath et al., 1999). Mn is essential for growth and fertility and plays a very important role in bone development in the embryo and after hatching in birds (Gallup and Norris, 1939; Fawcett, 1994; Underwood and Suttle, 2001).

Historically, animal feeds have been supplemented with low cost, inorganic forms of Zn, Cu, and Mn, such as oxides and sulfates. However, these inorganic salts tend to dissociate in the low pH environment of the upper gastrointestinal tract, leaving the minerals susceptible to reaction with other compounds that might impair absorption thereby decreasing mineral bioavailability and increasing mineral excretion and oxidative stress (Underwood and Suttle, 2001). Organic trace mineral (OTM) varies between products and are determined by the type of ligands used to bind with the mineral. Mineral–organic ligand combinations can result in the production of one of several classes of OTMs. These ligands are usually amino acids, peptides, polysaccharides and organic acids (Byrne et al., 2021). In general, organic microminerals are absorbed anywhere in the small intestine by intestinal carriers of amino acids and peptides while inorganic metals are generally in duodenum by intestinal transporters, so competition between minerals for the same absorption mechanism is reduced. Therefore, not only is the bioavailability of nutrients higher, but additionally minerals in organic form are readmitted to the tissues, and kept stored for a longer time compared to inorganic minerals (Lopes, et al., 2017). It should also be noted that not all organic trace mineral forms are equally stable at low pH and consequently the bioavailability of nutrients can be variable (Brown and Zeringue, 1994; Cao et al., 2000; Guo et al., 2001). Since organic trace minerals have greater bioavailability than inorganic trace minerals, the bis-chelated trace minerals, MMHAC, are chelated to a hydroxy methionine (MHA) analog, which is not an amino acid, but an organic acid. MHA is the precursor of methionine and differs from methionine by having a hydroxyl group instead of an amino group on the alpha carbon. The bis-chelates form 2 structural rings, which provide a higher protection for the metal in challenging environments such as the upper gastro-intestinal tract. This results in a much smaller dissociation rate versus other structures, such as the organic metal amino acid complexes or proteinates. MMHAC has been shown to be highly bioavailable with higher metallothionein synthesis in the enterocytes and/or greater metal deposition in bones than is seen with inorganic trace minerals (Yan and Waldroup, 2006; Richards et al., 2015; Min et al., 2019). Numerous studies have shown organic trace minerals to improve characteristics such as production performance, egg quality, reproduction, antioxidant status, mineral bioavailability and fecal mineral excretion in breeders, and bone development in broilers (Araújo et al., 2019; Wang et al., 2019a, 2019b; Güz et al., 2019; Londero et al., 2020). However, there are few studies evaluating the effects of feeding lower levels of organic trace minerals on breeder development and progeny growth and development. Therefore, the aim of this study was to evaluate the effect of two different organic trace minerals at low levels compared with inorganic trace minerals at high levels on performance (starter, growth and pre-reproduction, and reproduction stages), egg quality, gut health and bone development of broiler breeders from 1 day to 66 weeks of age and on progeny growth and development.

## Materials and methods

### Animals and management

The project was approved by the Ethics Committee on animal use (CEUA) of the Faculty of Animal Science and Food Engineering (FZEA) of the University of São Paulo. The study was conducted over a total period of 66 weeks between April 2019 and September 2020 at the Poultry Research Laboratory of the Department of Nutrition and Animal Production of the Faculty of Veterinary Medicine and Animal Science (FMVZ), Fernando Costa campus, Pirassununga, São Paulo - Brazil.

A total of 540 females 1-day old Cobb 500 (Slow feathering) broiler breeders with a uniform body weight (47.35 ± 0.013 g) were randomly assigned to three treatment groups with 15 replicates/treatment and 12 birds/replicate. The three treatment groups were: ITM-inorganic trace minerals in sulfates: 100, 16, and 100 ppm of Zn, Cu, and Mn respectively; MMHAC - mineral methionine hydroxy analog chelate: 50, 8, and 50 ppm of MINTREX^®^Zn, Cu, and Mn (Novus International, Inc.), respectively, and TMAAC - trace minerals amino acid complex: 50, 8, and 50 ppm of Zn, Cu, and Mn, respectively.

Feeding, water and lighting program were performed as recommended by genetic company, following a daily program the birds were fed *ad libitum* until the 7th day of age and feed intake was then controlled to ensure the birds reached sexual maturity at the appropriate age and feed intake of all birds was controlled as recommended by management guide. From the 1st to the 24th week, feed supply was adjusted weekly according to the management guide of the genetics company (Cobb, 2020). From the 24th to 66th week, the birds were weighed every 2 weeks to minimize the stress experienced by breeders in the laying phase.

In order for the breeding flock to have good uniformity, it is important to ensure that all birds have the same weight. In the present study, selection of breeding stock was performed twice. One way to accomplish this is to separate overweight and underweight animals from the average, ideal weight at 4 weeks of age, sort and monitor body weight during the growth period. This was followed by repeated sorting at 10 weeks of age, handling all males and removing suboptimal males with visual defects including crooked and bent fingers, abnormalities and bent toes, spinal abnormalities, eye and beak abnormalities. At 4 weeks, 3 birds per box were removed from the study and at 18 weeks another 2 birds per box were removed from the study, according to their body weight (±10% of the reference weight average).

### Diet and experimental groups

Basal diets that contained corn, soybean meal, wheat bran and trace minerals (Tables 1, 2) were formulated according to the nutritional requirement recommended in the guide from the genetics company (Cobb, 2020). The feeding program was divided into 6 phases: Starter (0–4 weeks), grower (5–18 weeks), pre-reproduction (19–22 weeks), reproduction I (23–40 weeks), reproduction II (41–50 weeks) and reproduction III (51–66 weeks).

**TABLE 1:** Composition of the basal diet for all stages of development.

Ingredients (g/kg)	Starter	Grower	Pre-reproduction	Reproduction I	Reproduction II	Reproduction III
Corn, 8.8%CP	58.680	59.320	64.740	70.940	70.880	70.640
Soybean meal., 45% CP	28.380	14.250	17.760	19.340	19.000	19.020
Soybean oil	3.210					
Wheat bran	5.000	21.860	10.840			
Premix*	2.000	2.000	2.000	2.000	2.000	2.000
Dicalcium phosphate	1.810	1.430	1.750	1.760	1.550	1.390
Limestone^a^	0.190	0.530	2.510	2.630	2.960	3.150
Salt	0.410	0.370	0.270	0.260	0.270	0.270
Sodium bicarbonate				0.150	0.150	0.150
MHA-methionine^b^	0.190	0.120	0.110	0.150	0.120	0.120
L-Lysine HCl. 78%	0.110	0.060		0.030	0.010	0.010
L-Threonine. 98%	0.020	0.060	0.020	0.010		
Zinc bacitracin. 15%				0.100	0.100	0.100
Total	100.0	100.0	100.0	100.0	100.0	100.0
Calculated composition, %						
AME^c^. Kcal/kg	2950	2763	2820	2820	2800	2780
Crude protein	19.000	15.360	15.500	15.000	14.500	14.000
Lysine disp	0.920	0.640	0.630	0.660	0.630	0.630
AAS disp	0.690	0.540	0.540	0.570	0.542	0.542
Methionine disp	0.490	0.290	0.280	0.310	0.380	0.380
Threonine disp	0.640	0.520	0.520	0.500	0.473	0.473
Isoleucine	0.720	0.510	0.520	0.500	0.549	0.549
Leucine	1.510	0.820	0.820	0.740	0.730	1.297
Tryptophane	0.210	0.150	0.160	0.170	0.157	0.157
Valine	0.770	0.470	0.490	0.530	0.504	0.504
Calcium	1.000	1.000	1.500	2.900	3.100	3.200
Phosphorus disp	0.450	0.400	0.440	0.420	0.380	0.350
Phosphorus total	0.790	0.800	0.670	0.610	0.670	0.670
Chlorine	0.280	0.260	0.200	0.190	0.196	0.196
Potassium	0.740	0.630	0.640	0.640	0.563	0.563
Sodium	0.200	0.180	0.180	0.180	0.180	0.180
Ac. linoleic	3.130	1.570	1.720	1.500	1.492	1.630

*Vitamin levels supplied in the feed through the premixes (considering inclusion of 20 kg/Ton): Vitamin A, 12000000 IU; Vitamin D3, 3,000,000 IU; Vitamin E, 75,000 IU; Vitamin K, 6,000 mg/ton; Thiamine, 2500 mg/ton; Riboflavin, 10,000 mg/ton; Pantothenic acid, 25,000 mg/ton; Niacin, 40,000 mg/ton; Pyridoxine, 6,000 mg/ton; Folic acid, 4,000 mg/ton; Vitamin B12, 35 mg/ton; Biotin, 300 mg/tone; Vitamin C, 50,000 mg/ton; Choline, 300,000 mg/ton.

a Limestone. 50% small particle size/50% Large particle size.

b MHA (84% methionine. 12% Ca. PB, 49.3%. EM, 4.014 kcal/kg).

c Apparent Metabolic Energy.

Disp: Disponible.

**TABLE 2:** Concentrations of zinc (Zn) Copper (Cu) and Manganese (Mg) in mineral premix at inclusion 20 kg/MT.

		Analyzed			Formulated		
		ITM	MMHAC	TMAAC	ITM	MMHAC	TMAAC
Zn	mg/kg	4516	2829	2614	5000	2500	2500
Cu	mg/kg	779	451	474	800	400	400
Mn	mg/kg	5702	2735	2703	5000	2500	2500

Value based on duplicate determinations; ITM—inorganic trace minerals in sulfates: 100, 16, and 100 ppm of Zn, Cu, and Mn respectively; MMHAC-mineral methionine hydroxy analog chelate: 50, 8, 50 ppm of bis-chelated MINTREX®Zn, Cu, and Mn (Novus International, Inc.), and TMAAC—trace minerals amino acid complex: 50, 8, and 50 ppm of Zn, Cu, and Mn.

### Evaluation of live performance

The growth and development of the broiler breeders was evaluated from 0 to 24 weeks. At the end of each phase (4; 10; 18, and 24 weeks), the birds were evaluated individually for the following characteristics: average body weight (kg), average daily body weight gain (g/bird/day), average feed intake (g) and corrected feed conversion ratio (FCR) (Sakomura and Rostagno, 2016).

At 24 weeks old, the birds started the production phase. The productive performance of the birds was evaluated daily after the stabilization of laying (26 weeks) and a cycle of (26–40, 26–50, and 26–66) was considered. The characteristics evaluated included daily egg production (percentage), daily egg mass (g) and FCR per egg mass. The data obtained were corrected if there was mortality in the group. At 28 weeks, the broiler breeder hens were inseminated with 0.5 ml fresh semen, which was collected by means of abdominal massaging. The eggs were collected from day 3 until day 10 after insemination and placed temporarily in a holding room at 18°C. Subsequently, the eggs from the broiler breeders were incubated in a commercial incubator with automatic turning. Incubation was performed at a temperature of 37.7°C and with relative air humidity of 55%–60%. The egg was turned automatically every hour through an angle of 45°.

### Progeny trial

From the hatching eggs of the breeders, a total of 360-day old chicks (180 males and 180 females) were used for part of the study. They were distributed according to their maternal diet in 10 pens with 6 males and 6 female birds per pen for a period of 45 days of age. Progeny received the same basal diet (Table 3) to investigate the effect of nutrient transfer from breeders to progeny.

**TABLE 3:** Composition of the basal diet for all stages of development of progeny broiler.

Ingredients (g/kg)	Starter (1–14 days)	Grower (15–28 days)	Finisher (29–45 days)
Corn 8.8% CP	61.911	68.546	69.857
Soybean meal 45% CP	33.410	27.200	25.420
Soybean oil	0.947	0.874	1.384
Premix*	0.500	0.500	0.500
Dicalcium phosphate	1.169	0.972	0.990
Limestone^a^	0.952	0.857	0.861
Salt	0.281	0.268	0.249
Sodium bicarbonate	0.201	0.186	0.177
MHA-Methionine^b^	0.306	0.265	0.248
L-Lysine HCl. 78%	0.223	0.249	0.238
L-Threonine. 98%	0.042	0.025	0.018
Salinomycin 12%	0.055	0.055	0.055
Phytaverse	0.003	0.003	0.003
Total	100.0	100.0	100.0
Calculated composition %			
AME^c^. Kcal/kg	2965	3050	3100
Crude protein	19.000	15.360	17.793
Lysine disp	0.920	0.640	0.630
AAS disp	0.690	0.540	0.540
Methionine disp	0.490	0.290	0.280
Threonine disp	0.640	0.520	0.520
Isoleucine	0.720	0.510	0.520
Leucine	1.510	0.820	0.820
Tryptophane	0.210	0.150	0.160
Valine	0.770	0.470	0.490
Calcium	1.000	1.000	1.500
Phosphorus disp	0.450	0.400	0.440
Phosphorus Total	0.790	0.800	0.670
Chlorine	0.280	0.260	0.200
Potassium	0.740	0.630	0.640
Sodium	0.200	0.180	0.180
Ac. Linoleic	3.130	1.570	1.720

*Vitamin levels supplied in the feed through the premixes (considering inclusion of 5 kg/Ton): Vitamin A, 2016.0 IU; Vitamin D3, 630.0 IU/g; Vitamin E, 10000.1 IU; Vitamin K, 630 mg/Ton; Thiamine, 756 mg/kg; Riboflavin, 1,512 mg/kg; Pantothenic acid, 2,520 mg/kg; Niacin, 10,080 mg/kg; Pyridoxine, 816 mg/kg; Folic acid, 189 mg/kg; Vitamin B12, 4536.1 mcg/kg; Selenium, 70 mg/kg Iodine, 200 mg/kg; Iron 8,000 mg/kg; Choline, 80 g/tkg.

1 Limestone. 100% small particle size.

2 MHA (84% methionine. 12% Ca. PB 49.3%. EM 4.014 kcal/kg).

3 Apparent Metabolic Energy.

Disp, disponible.

The broiler diets were based on corn and soybean meal with supplementation of ITM—inorganic trace minerals: 100, 16, and 100 ppm of Zn, Cu, and Mn respectively, as sulfates. The feeding program was divided into 3 phases: starter (1–14 days); grower (15–28 days) and finisher (29–45 days). Water and feed were provided *ad libitum*. At the end of each phase, the following performance evaluations were measured: body weight (BW), daily body weight gain (DBWG), daily feed intake (DFI), FCR and performance index (PEI) (Bird, 1955).

### Egg quality

Egg quality was evaluated at 28, 40, 50, 58, and 65 weeks of age. For two consecutive days, a total 120 eggs per treatment were collected for analysis, identified and, the egg weight (g), yolk coloration, eggshell thickness (mm) and resistance (kgf) were measured using the Digital Egg Tester machine (DET6000, Nabel Co.). For egg quality data, the average of the eggs laid by each experimental unit (box) within the two consecutive days was taken.

### Gut health

To determine whether trace minerals affect gut health, expression of nuclear factor kappa B (NF-kB) was measured in the jejunum of 15 breeders per treatment (1 breeder per box) at 24°weeks of age, expression of NF-kB, interleukin 6 (IL6), mucin-2 protein (MUC2), interleukin-1β (IL-1β) and toll like receptor 2 (TLR2) was measured in 10 progeny broilers per treatment (1 bird per pen) at 1, 14, and 45 days of age by qRT-PCR. For extraction of genetic material from the jejunum, the 2-cm segments of jejunum were cut down to Meckel’s diverticulum, then the tissue was carefully opened with sterile scissors (70% alcohol) and very gently cleaned with cold sterile PBS. It was then placed in an aluminum foil, which was closed quickly and immediately and placed into liquid nitrogen for at least 24 h and kept in a freezer (−80°C) until analysis.

Total RNA was extracted from jejunal tissue using the Trizol^®^ (Invitrogen - Thermo Fisher Scientific, Waltham, Massachusetts, United States). Samples were treated with DNaseI (Invitrogen-Thermo Fisher Scientific, Waltham, Massachusetts, United States) and reverse transcription was carried out with High-Capacity cDNA Reverse Transcription Kits (Applied Biosystems - Thermo Fisher Scientific, Waltham, Massachusetts, United States).

The RT-qPCR analysis was conducted using SYBR^®^ Green PCR Master Mix (Applied Biosystems - Thermo Fisher Scientific, Waltham, Massachusetts, United States) on the Step One Plus Real-Time PCR System (Applied Biosystems- Thermo Fisher Scientific, Waltham, Massachusetts, United States). Primers were designed using OligoAnalyzer 3.1 (Integrated DNA Technologies, Coralville, Iowa, United States) (Table 4) and purchased from Invitrogen (Thermo Fisher Scientific, Waltham, Massachusetts, United States). The concentration of primers was optimized and primers were verified with 95%–105% efficiency and linearity of amplification *r* ^*2*^ greater than 0.99. Relative fold change in mRNA quantity was normalized to house-keeping gene β-actin and calculated according to 2-ΔΔCT (Livak and Schmittgen, 2001), and the relative expression of all genes were then normalized to ITM treatment.

**TABLE 4:** The sequence of primers for qRT-PCR.

Gene	Forward primer sequence (5′–3′)	Reverse primer sequence (5′–3′)	Accession number	Fragment legnth (bp)
β-actin	CAA​CAC​AGT​GCT​GTC​TGG​TGG​TA	ATC​GTA​CTC​CTG​CTT​GCT​GAT​CC	L08165	205
IL6	GAG​GGC​CGT​TCG​CTA​TTT​G	ATT​GTG​CCC​GAA​CTA​AAA​CAT​TC	AJ309540	63
A20	AGG​CTC​CTC​CTG​TGG​TAA​AGC	GAA​AAG​GCT​GGG​AGC​AGT​TG	XM_003640919.2	99
NF-κB	GTG​TGA​AGA​AAC​GGG​AAC​TG	GGC​ACG​GTT​GTC​ATA​GAT​GG	NM_205129	202
MUC2	GCC​TGC​CCA​GGA​AAT​CAA​G	CGA​CAA​GTT​TGC​TGG​CAC​AT	BX930545	59
IL-1β	CAGCCCGTGGGCATCA	CTT​AGC​TTG​TAG​GTG​GCG​ATG​TT	NM_204524	58
TLR2	CGC​TTA​GGA​GAG​ACA​ATC​TGT​GAA	GCC​TGT​TTT​AGG​GAT​TTC​AGA​GAA​TTT	NM_001161650.3	90

IL6, interleukin 6; A20, alpha-induced protein 3; NF-kB, nuclear factor kappa B; MUC2, mucin-2, protein; IL-1β, interleukin-1β; TLR2, toll like receptor 2.

### Bone quality

Bone quality was evaluated in 15 breeders per treatment (1 breeder per box) at 18, 24, and 66 weeks of age. The right tibia was removed from the birds slaughtered for evaluation of wet weight (WW), dry weight (DW), dry matter (DM), length (LGTH), circumference (CIR) and resistance (R) were measured. For bone density, the Seedor index (bone weight/bone length) was calculated.

The tibias were desiccated to determine bone resistance using a universal EMIC^®^ test machine, model DL 3000, with load applied at a speed of 5 mm/min and a load cell of 2000 N. In the three-point flexion test, the tibias were held in the horizontal position with support span distance of 3/4 the size of the bone and the force was applied at a point half way between the device and the tibia.

Dry matter of the tibia was evaluated using the methodology described by the Association of Official Analytical Chemists (AOAC, 2007). The right tibia was deboned. The weight of the clean crucible (g) and the weight of the sample (g) were measured. Then, the crucibles with the sample were placed in a forced ventilation oven at 105°C for 24 h. The dry matter weight of the sample was determined to be the difference between final weight of sample and crucible less the weight of the crucible. The quotient was multiplied by 1,000 for conversion between mass units (g and kg).

### Statistical analysis

The results were analyzed and tested for normality. Observations with a studentized residual greater than three (in absolute value) were considered outliers.

The data were submitted to one way ANOVA analysis and the means of treatments compared by Tukey test using the MIXED PROC of the Statistical Analysis System (SAS) software (Version 9.4, 2013). A *p* value ≤ 0.05 was considered as significant and 0.05 < *p* value ≤ 0.1 was considered as a trend.

## Results

### Performance of broiler breeders during rearing phase

There was no significant difference in average body weight (BW), daily body weight gain (DBWG) and feed conversion (FCR) between treatments except for the period 0–10 weeks (grower phase) (Table 5). During the grower phase, BW and DBWG were lower for the breeders in MMHAC than those in ITM but not different from TMAAC. FCR was better in the ITM group than MMHAC but there was no difference between ITM and TMAAC groups. There was no significant difference in daily feed intake (DFI) between any groups in the evaluated periods.

**TABLE 5:** Effect of inorganic or organic trace minerals on the performance of broiler breeders.

Trace mineral source	BW, kg	DBWG, g	DFI, g	FCR
0–4 weeks				
ITM	0.555	18.134	33.699	1.851
MMHAC	0.554	18.098	33.724	1.865
TMAAC	0.554	18.085	33.693	1.856
SEM	0.009	0.084	0.060	0.051
*p* value	0.8860	0.9172	0.9291	0.7518
0–10 weeks				
ITM	1.178^a^	16.151^a^	43.779	2.545^b^
MMHAC	1.150^b^	15.761^b^	43.787	2.608^a^
TMAAC	1.161^ab^	15.909^ab^	43.777	2.596^ab^
SEM	0.024	0.090	0.024	0.065
*p* value	0.0130	0.0132	0.9521	0.0372
0–18 weeks				
ITM	1.973	15.279	54.100	3.340
MMHAC	1.970	15.257	54.104	3.354
TMAAC	1.970	15.258	54.099	3.346
SEM	0.047	0.113	0.013	0.080
*p* value	0.9868	0.9883	0.9653	0.9258
0–24 weeks				
ITM	3.100	18.173	68.032	3.543
MMHAC	3.060	17.928	68.037	3.601
TMAAC	3.095	18.145	68.032	3.567
SEM	0.076	0.141	0.010	0.082
*p* value	0.4214	0.4122	0.9309	0.3987

ab Means within a column without a common superscript differ (*p* ≤ 0.05).

ITM, inorganic trace minerals: 100, 16, and 100 ppm of Zn, Cu, and Mn respectively, as sulfates; MMHAC-mineral methionine hydroxy analog chelate: 50, 8, 50 ppm of bis-chelated MINTREX®Zn, Cu, and Mn (Novus International, Inc.), and TMAAC—trace minerals amino acid complex: 50, 8, and 50 ppm of Zn, Cu, and Mn.

Body weight (BW), daily body weight gain (DBWG), daily feed intake (DFI) and feed conversion (FCR).

### Performance of broiler breeders in the reproduction phase

There was no significant difference in egg production (%) and FCR per egg mass (Table 6).

**TABLE 6:** Effect of feeding supplemental inorganic or organic trace minerals on the performance of broiler breeders (26–66 weeks).

Trace mineral source		Egg production, %	Egg mass, g	FCR per egg mass	Egg number
26–66 weeks					
	ITM	63.9	41.9^b^	3.96	169.14
	MMHAC	67.9	44.9^a^	3.62	180.03
	TMAAC	66.2	44.0^ab^	3.76	175.58
	SEM	1.6	1.2	0.14	4.35
*p* value	Treatment	0.2036	0.0262	0.0987	0.2100

ab Means within a column without a common superscript differ (*p* ≤ 0.05).

ITM, inorganic trace minerals: 100, 16, and 100 ppm of Zn, Cu, and Mn respectively, as sulfates; MMHAC-mineral methionine hydroxy analog chelate: 50, 8, 50 ppm of bis-chelated MINTREX®Zn, Cu, and Mn (Novus International, Inc.), and TMAAC—trace minerals amino acid complex: 50, 8, and 50 ppm of Zn, Cu, and Mn.

For egg mass, a significant difference (*p* = 0.0262) was observed between treatments at 26–66 weeks (Table 6). MMHAC improved egg mass vs. ITM during weeks 26–66 (*p* = 0.0262, Table 6).

### Egg quality

There was no significant difference in egg weight at any time points (Table 7). MMHAC supplementation enhanced (*p* < 0.009) yolk coloration in comparison to both ITM and TMAAC at all time points except for week 28, improved eggshell resistance vs. TMAAC at week 50 (*p* = 0.0913) and week 58 (*p* = 0.0027), decreased (*p* = 0.0196) eggshell thickness vs*.* ITM but not different from TMAAC at week 28 and increased (*p* = 0.002) eggshell thickness vs. TMAAC at week 58 (Table 7).

**TABLE 7:** Effect of inorganic or organic trace minerals on egg quality parameters of broiler breeders.

Trace mineral source	Weight (g)	Yolk coloration	Resistance (kgf)	Eggshell thickness (mm)
Week 28				
ITM	57.178	8.617	4.103	0.393^a^
MMHAC	57.944	8.523	3.974	0.383^b^
TMAAC	58.152	8.52	4.093	0.379^b^
SEM	0.455	0.080	0.080	0.003
*p* value	0.2916	0.6191	0.4498	0.0196
Week 40				
ITM	66.708	6.550^b^	4.058	0.398
MMHAC	67.404	6.922^a^	3.85	0.398
TMAAC	67.484	6.598^b^	3.784	0.39
SEM	0.528	0.080	0.102	0.004
*p* value	0.5225	0.0036	0.1535	0.3271
Week 50				
ITM	68.028	5.905^b^	3.767	0.369
MMHAC	69.058	6.182^a^	3.764	0.375
TMAAC	70.178	6.000^b^	3.520	0.377
SEM	0.819	0.061	0.089	0.005
*p* value	0.1835	0.0082	0.0913	0.5045
Week 58				
ITM	70.707	6.595^b^	3.594^a^	0.398^a^
MMHAC	72.372	7.161^a^	3.593^a^	0.394^a^
TMAAC	73.005	6.479^b^	3.030^b^	0.372^b^
SEM	0.915	0.074	0.123	0.005
*p* value	0.2044	< 0.0001	0.0027	0.0020
Week 65				
ITM	72.146	7.991^b^	3.293	0.379
MMHAC	73.604	8.331^a^	3.512	0.374
TMAAC	73.155	7.548^c^	3.408	0.375
SEM	0.738	0.088	0.120	0.005
*p* value	0.3674	< 0.0001	0.4357	0.7839

abc Means within a column without a common superscript differ (*p* ≤ 0.05).

ITM—inorganic trace minerals: 100, 16, and 100 ppm of Zn, Cu, and Mn respectively, as sulfates; MMHAC-mineral methionine hydroxy analog chelate: 50, 8, 50 ppm of bis-chelated MINTREX®Zn, Cu, and Mn (Novus International, Inc.), and TMAAC—trace minerals amino acid complex: 50, 8, and 50 ppm of Zn, Cu, and Mn.

### Gut health of broiler breeders

**FIGURE 1:**
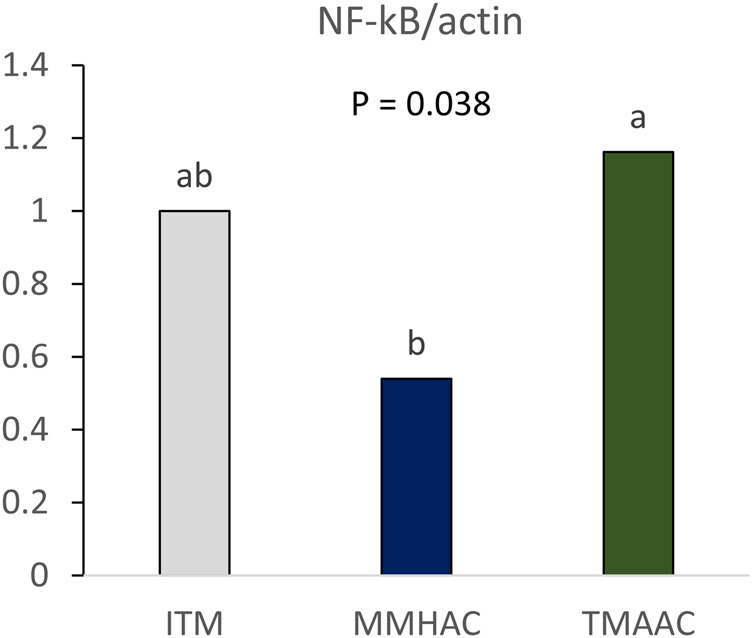
Effect of inorganic or organic trace minerals on jejunal NFκB gene expression in broiler breeders at 24 weeks of age. ^ab^ Means without a common superscript differ (*p* ≤ 0.05). ITM—inorganic trace minerals: 100, 16, and 100 ppm of Zn, Cu, and Mn respectively, as sulfates; MMHAC-mineral methionine hydroxy analog chelate: 50, 8, 50 ppm of bis-chelated MINTREX–Zn, Cu, and Mn (Novus International, Inc.), and TMAAC—trace minerals amino acid complex: 50, 8, and 50 ppm of Zn, Cu, and Mn.

To determine whether trace minerals affect gut health, NF-κB mRNA expression was measured in the jejunum of breeders at 24 weeks of age. In the groups with diets supplemented with MMHAC, NF-κB mRNA expression was significantly lower than TMAAC and numerically lower than ITM (Figure 1), indicating that supplementation with MMHAC reduced jejunal inflammation in broiler breeder.

### Bone quality

At 18 weeks of age, there was no significant difference between treatments for wet weight (WW), length and circumference of the tibia (Table 8). Dry weight, dry matter, Seedor index and resistance were significantly reduced in MMHAC treatment when compared to ITM, but not different from TMAAC.

**TABLE 8:** Effect of feeding supplemental inorganic or organic trace minerals on bone quality.

Trace mineral source	WW, %	DW, %	DM, %	LGTH, cm	Seedor Índex	CIR, mm	R, kg/N
18 weeks							
ITM	16.22	10.65^a^	65.79^a^	12.56	8.48^a^	7.87	7.28^a^
MMHAC	15.59	9.54^b^	61.49^b^	12.50	7.64^b^	7.87	4.86^b^
TMAAC	15.98	10.04^b^	62.79^ab^	12.60	7.96^b^	8.11	5.54^b^
SEM	0.35	0.22	1.02	0.12	0.14	0.09	0.38
*p* values	0.4534	0.0034	0.0146	0.8550	0.0006	0.1177	<0.0001
24 weeks							
ITM	19.86	12.56	63.24	12.63	9.94^a^	8.17	12.94
MMHAC	19.67	11.99	61.17	12.73	9.41^b^	8.41	11.73
TMAAC	19.79	12.50	63.36	12.63	9.97^a^	8.18	13.62
SEM	0.46	0.24	0.85	0.09	0.17	0.08	0.72
*p* values	0.9580	0.2000	0.1315	0.6363	0.0399	0.0920	0.1797
66 weeks							
ITM	26.92	16.39	60.93	12.56	12.96	8.34^b^	11.20^a^
MMHAC	26.71	16.83	63.14	12.35	13.45	8.10^b^	9.19^ab^
TMAAC	26.36	16.07	61.03	12.55	12.82	8.81^a^	8.79^b^
SEM	0.53	0.37	0.94	0.12	0.29	0.09	0.59
*p* values	0.7600	0.3500	0.1800	0.3572	0.2408	<0.0001	0.0133

ab Means within a column without a common superscript differ (*p* ≤ 0.05).

ITM, inorganic trace minerals: 100, 16, and 100 ppm of Zn, Cu and Mn respectively, as sulfates; MMHAC-mineral methionine hydroxy analog chelate: 50, 8, 50 ppm of bis-chelated MINTREX®Zn, Cu, and Mn (Novus International, Inc.), and TMAAC—trace minerals amino acid complex: 50, 8, and 50 ppm of Zn, Cu, and Mn.

Wet weight (WW), dry weight (DW), dry matter (DM), length (LGTH), circumference (CIR) and resistance (R).

At 24 weeks of age, MMHAC significantly (*p* = 0.0399) reduced seeder index and numerically (*p* = 0.092) increased tibial circumference in comparison to ITM and TMAAC. There were no significant differences in other tibial parameters between treatments.

At the end of the study, 66 weeks, MMHAC supplementation did not significantly alter bone characteristic parameters in comparison with ITM and TMAAC except for a lower (*p* < 0.0001) tibial circumference and numerically higher tibial resistance than TMAAC. Although TMAAC treatment resulted in a higher tibial circumference than ITM and MMHAC, tibial resistance was lower (*p* = 0.0133) than ITM.

### Performance of the progeny

The body weight of progeny chicks at hatch was significantly higher in MMHAC treatment than TMAAC and ITM treatments (Table 9). During starter phase (1–14 days), there was no significant difference in BW, DBWG, FCR, and PEI between treatments, however, FI was significantly (*p* = 0.0231) reduced in MMHAC when compared to ITM, but not different from TMAAC (Table 9).

**TABLE 9:** Effect of inorganic or organic trace minerals on the performance of progeny.

Trace mineral source	BW, g	BWG, g	DBWG, g	FI, g	FCR	PEI
At hatch (day 1)						
ITM	42.000^c^					
MMHAC	43.860^a^					
TMAAC	42.940^b^					
SEM	0.070					
*p* value	<0.0001					
0–14 days						
ITM	408.929	366.929	26.209	536.250^a^	1.401	181.072
MMHAC	390.231	346.374	24.741	488.651^b^	1.435	170.482
TMAAC	398.507	355.564	25.397	483.291^b^	1.385	175.413
SEM	13.406	13.387	0.956	13.989	0.023	9.738
*p* value	0.6188	0.5604	0.5604	0.0231	0.2854	0.7460
0–28 days						
ITM	1269.470	1227.470	43.838	1926.667^a^	1.541^ab^	279.697^a^
MMHAC	1215.125	1171.268	41.831	1726.170^b^	1.484^b^	277.041^a^
TMAAC	1181.795	1138.852	40.673	1832.942^ab^	1.612^a^	239.674^b^
SEM	30.721	30.703	1.097	43.803	0.028	11.056
*p* value	0.1450	0.1380	0.1380	0.0118	0.0051	0.0262
0–45 days						
ITM	2853.488	2811.488	62.478	4877.947	1.728	357.521
MMHAC	2807.759	2763.902	61.42	4615.985	1.695	352.832
TMAAC	2767.417	2724.474	60.544	4678.027	1.720	334.144
SEM	43.901	43.891	0.975	79.013	0.021	8.420
*p* value	0.3947	0.3862	0.3862	0.0665	0.5047	0.1351

abc Means within a column without a common superscript differ (*p* ≤ 0.05).

ITM, inorganic trace minerals: 100, 16, and 100 ppm of Zn, Cu and Mn respectively, as sulfates; MMHAC-mineral methionine hydroxy analog chelate: 50, 8, 50 ppm of bis-chelated MINTREX®Zn, Cu, and Mn (Novus International, Inc.), and TMAAC—trace minerals amino acid complex: 50, 8, and 50 ppm of Zn, Cu, and Mn.

Body weight (BW), body weight gain (BWG), daily body weight gain (DBWG), feed intake (FI), feed conversion ratio (FCR) and Performance index (PEI).

During 1–28 days, maternal supplementation of MMHAC significantly (*p* = 0.0118) reduced FI vs. ITM and improved FCR (*p* = 0.0051) and PEI (*p* = 0.0262) vs. TMAAC (Table 9).

Considering performance characteristics across the entire period, 0–45 days, BW, DBWG, FCR and PEI were not different between treatments, FI was numerically (*p* = 0.0665) reduced in MMHAC and TMAAC treatments compared with ITM.

### Gut health of progeny

**FIGURE 2:**
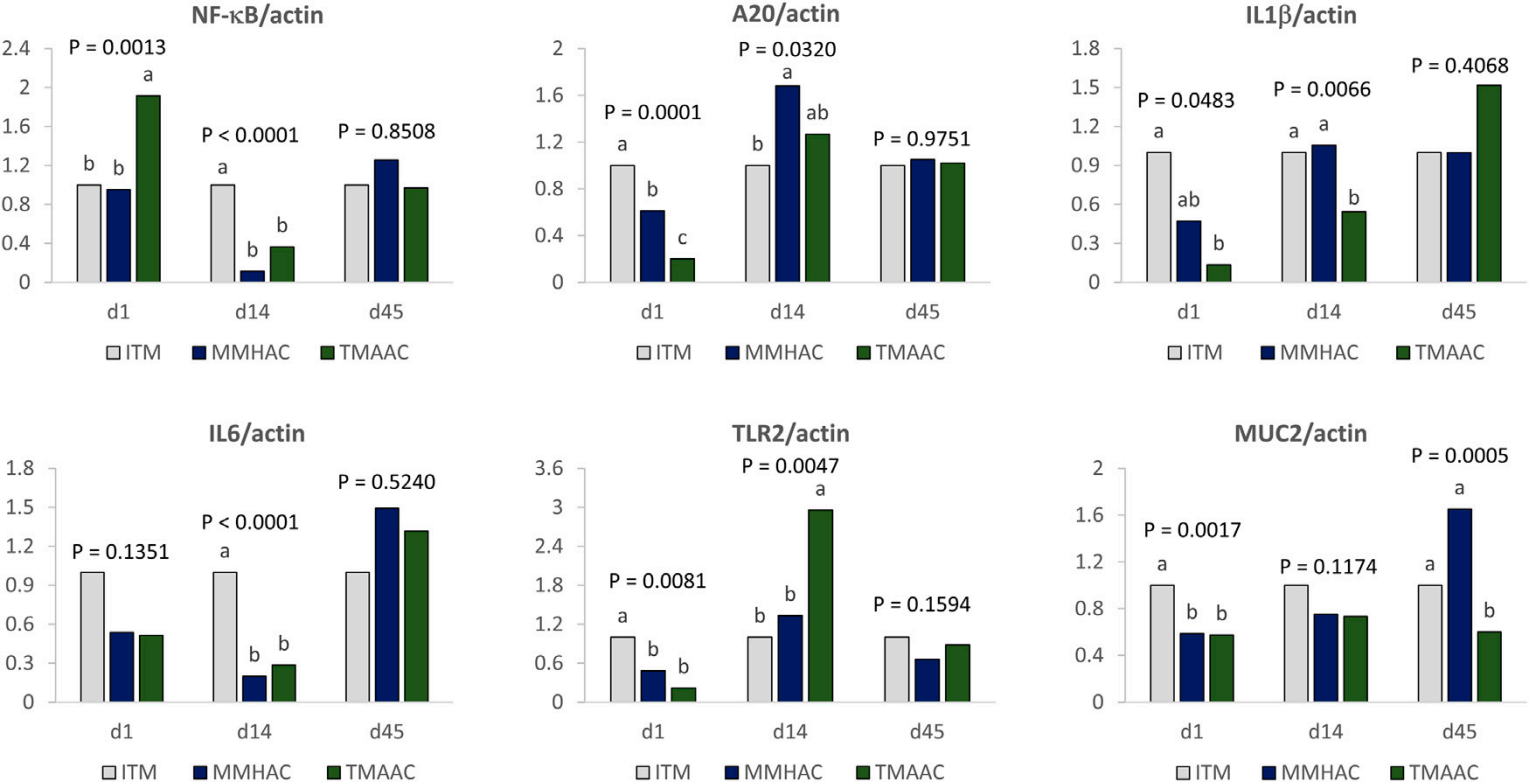
Effect of inorganic or organic trace minerals on jejunal anti-inflammatory gene expression in progeny broilers at 1-, 14-, and 45-days of age. ^ab^ Means without a common superscript differ (*p* ≤ 0.05). ITM—inorganic trace minerals: 100, 16, and 100 ppm of Zn, Cu and Mn respectively, as sulfates; MMHAC-mineral methionine hydroxy analog chelate: 50, 8, 50 ppm of bis-chelated MINTREX®Zn, Cu, and Mn (Novus International, Inc.), and TMAAC—trace minerals amino acid complex: 50, 8, and 50 ppm of Zn, Cu, and Mn.

To determine whether trace minerals in the maternal diet affect gut health of the progeny, MUC2, IL6, NF-κB, IL1β, A20, and TLR2 mRNA expression was measured in the jejunum of progeny at 1, 14, and 45 days of age (Figure 2).

At hatch, in the groups with maternal diets supplemented with MMHAC, TLR2, and MUC2 mRNA expression was lower than ITM but not different from TMAAC, NF-κB mRNA expression was significantly lower than TMAAC but no different from ITM, A20 gene expression was lower than ITM and higher than TMAAC (Figure 2).

At the end of starter stage, 14 days of age, MMHAC maternal supplementation resulted in significantly (*p* < 0.0001) lower IL6 and NF-κB mRNA expression for the progeny in comparison with ITM but there was no difference from TMAAC. Progeny from maternal breeders with MMHAC supplementation also had higher IL1β and lower TLR2 expression than TMAAC, and higher A20 expression than ITM.

At the end of the study, at 45 days of age, the only gut health parameter significantly altered by maternal MMHAC supplementation in comparison with ITM and TMAAC was MUC2 mRNA expression. Maternal MMHAC supplementation significantly (*p* = 0.0005) increased MUC2 mRNA expression in comparison with ITM and TMAAC.

## Discussion

In this study, supplementation of low dose of MMHAC and TMAAC had similar development and growth performance as high dose of ITM in broiler breeders, and tended to improve egg production % and egg mass and FCR per egg mass as compared to high dose of ITM. MMHAC also improved egg yolk color in comparison to ITM and TMAAC at most time points and eggshell resistance and thickness vs. TMAAC at 58 weeks. These benefits are probably due to higher bioavailability of chelated Zn, Cu, and Mn-methionine hydroxy analogs than inorganic trace minerals as reported previously (Yan and Waldroup, 2006; Richards et al., 2015; Min et al., 2019).

Consistent with the findings in this study, a number of other studies have showed beneficial effects of chelated Zn-MHA and chelated Zn, Cu, and Mn-MHA in breeder reproduction performance and egg quality. For example, supplementation of 0.5 and 1 g/kg MMHAC analog and organic Se significantly improved egg production by 8.4–9.3% points, egg mass by 6.28–7.23 g of egg/hen/day and feed conversion ratio by 28–32 points in comparison to ITM in commercial laying hens raised under high ambient temperature. It also increased eggshell thickness and albumen width, reduced total cholesterol in serum and egg yolk and malondialdehyde in egg yolk, and increased antibody titers against avian influenza H9N1. This suggests that supplementation with MMHAC enhances reproductive performance, shell quality characteristics, plasma profile, yolk mineral concentration, yolk lipid oxidation, and immune response in laying hens under high ambient temperature (Saleh et al., 2020). Consistent with these findings, MMHAC supplementation in breeders over the 80 weeks produced a 4.1% increase in total egg production per hen housed (326 vs. 339) and a 4.9% increase in hatchable eggs per hen housed (119 vs. 124) vs. ITM (Parker, 2013). Li et al. (2019) reported that shell strength was mostly related to mammillary cone width in the ultrastructure, which is affected by the pattern of calcium deposition during eggshell formation. Furthermore, the supplementation of 80 mg/kg chelated Zn-methionine hydroxy analog in Hyline Grey layers significantly increased egg weight and eggshell strength (Li et al., 2019). This was probably caused by Zn-induced changes in Ca deposits in the eggshell. The addition of Zn increases the utilization of Ca to improve the qualitative parameters of the eggshell.

Similarly, Min et al. (2018) found that supplementation of 40 or 80 mg/kg chelated Zn-methionine hydroxy analog significantly increased eggshell thickness and eggshell strength by promoting Ca deposition and carbonic anhydrase activity and reduced the percentage of broken eggs in comparison to 80 mg/kg ZnSO_4_ (Min et al., 2018).

Araújo et al. (2019) observed that low levels of organic trace minerals Zn:Cu:Mn:Fe, in the form of carbo-amino-phosphates, increased egg production and eggshell thickness and breaking strength in broiler breeders at 34, 46, and 52 weeks of age when compared to high levels of inorganic trace minerals (Araújo et al., 2019). Wang et al., 2019a reported that organic trace minerals Zn, Cu, Mn, Fe, and Se, in the form of proteinate, increased laying rate by 9.56% in comparison to the same levels of ITM, but not with respect to high levels of ITM (Wang et al., 2019b). However, the same supplementation did not improve egg weight and egg quality vs. the same or higher levels of ITM (Wang et al., 2019a). Although Umar Yaqoob et al., 2020 observed no significant difference of the productive performance, higher eggshell thickness was seen when broiler breeders were fed low levels of organic trace minerals Zn, Cu, Mn, and Fe in the form of complexed glycinate in comparison to commercially recommended high levels of ITM during weeks 23–37 (Umar Yaqoob et al., 2020).

The influence of Zn supplementation levels on productive indices of laying hens is inconsistent. A meta-analysis from 11 studies showed that dietary organic and/or inorganic Zn supplementation reduced FCR and increased egg weight and egg mass compared with control diets without Zn supplementation (Ogbuewu and Mbajiorgu, 2022). The results of meta-analysis were affected by the age and breed/strain of hen, and supplementation levels and duration. In this meta-analysis, Zn source (organic vs. inorganic) had no effect on feed intake, but organic Zn significantly improved FCR, egg production%, egg quality (including egg weight, egg mass, Haugh unit, eggshell thickness, eggshell weight), and blood Zn concentrations in laying hens. Supplementation of 25, 50, 75 or 100 ppm of zinc methionine had no effect on body weight, body weight gain or feed conversion ratio in comparison to a control diet without Zn supplementation in Hisex Brown laying hens from 22 to 34 weeks of age, and 100 ppm of zinc methionine significantly increased egg number, egg weight and egg mass compared to other groups (Abd El-Hack et al., 2018). These findings suggest that organic Zn has greater bioavailability than inorganic Zn, leading to better nutrient digestibility and absorption in the gastrointestinal tract thereby greater reproduction performance.

The changes in breeder productivity with different forms of organic trace minerals may be related to the type of ligand on the mineral. In the present study, each molecule of Zn, Cu, and Mn was chelated to 2 molecules of methionine hydroxy analog by covalent bonds, which are far more stable than ionic bonds (Kratzer, 2017). The bis-chelate structure of MMHAC protects the minerals during transit through the gastrointestinal tract and ensures the mineral is delivered to the absorption site in the small intestine. The ligand, methionine hydroxy analog, is not an amino acid but an organic acid, 2-hydroxy-4-methyliobutanoic. It is the precursor of Met, and rapidly absorbed and converted into L-methionine in the bird (Dibner, 2003). Bis-chelated Zn has been shown to have greater bioavailability, with a higher metallothionein expression in the enterocytes or a higher amount of Zn deposition in bones, than other forms of organic Zn, such as Zn-Met, Zn proteinate and Zn amino acid complex (Richards et al., 2008a; Richards et al., 2008b). Supplementation of methionine hydroxy analog bis-chelated Zn to breeders also increased apparent retention of minerals such as Cu, Zn, and Ca and nutrients such as crude protein and energy, expression of liver metallothionein, tibial strength and tibial Zn and Ca deposition in aged hens (Min et al., 2019). The numerically higher egg production, egg mass, egg quality and better FCR per egg mass in MMHAC group is probably due to the greater bioavailability and nutrient retention of MMHAC than the TMAAC in this study.

It has been reported that the effect of maternal dietary organic trace mineral supplementation can be felt beyond the embryonic stage (Zorzetto et al., 2021). It is well recognized that the trace minerals Zn, Cu, and Mn could potentially impact optimal gut health of young monogastric animals (Shannon and Hill, 2019). Zn is known to be essential in many biological functions in mammals, such as anti-inflammation, anti-diarrhea, and maintenance of the integrity of the epithelial barrier (Roselli et al., 2003; Patel et al., 2010). Supplementation with zinc oxide with small particle size improved gut morphometry and gut barrier function in Ross 308 broiler breeder hens (Barzegar et al., 2021) and increased anti-sheep red blood cells antibody titer, IgM, the total number of leukocytes, and the percentage of lymphocytes in Cobb500 breeder hens (Sharideh et al., 2019). Adequate or higher maternal Zn supplementation to breeder hens not only improved breeder performance with 4%–7% higher laying rate, but also enhanced progeny immune response and antioxidant ability via epigenetic mechanisms (Huang et al., 2019). For example, supplementation of bis-chelated Zn-MHA in breeder hens improved progeny immunity by down-regulation of NF-kB mediated intestinal inflammation (Li et al., 2015). The NF-κB signaling pathway is one of the major pathways regulating chronic inflammation and inflammation induced by external stimuli such as bacteria, infection (Neurath et al., 1998). Zinc finger protein A20 is a signaling molecule that blocks the phosphorylation and activation of NF-κB, thereby inhibiting translocation of NF-κB and suppressing the inflammatory cascade and reducing expression of proinflammatory cytokines (Ma and Malynn, 2012; Catrysse et al., 2014). Therefore, upregulation of A20 would downregulate the NF-κB pathway and reduce inflammation. In the current study, MMHAC supplementation significantly reduced NF-κB mRNA expression vs*.* TMAAC in breeders and decreased NF-κB mRNA expression in progeny chicks vs*.* either ITM or TMAAC on d0 and d14, and upregulated A20 gene expression vs. ITM on d14. These results suggest that the downregulation of the NF-κB pathway in progeny birds might be caused by the upregulation of A20 gene expression. This is consistent with the study by Li et al. (2015), in which chelated Zn supplementation to breeder hens exhibited greater effects than ZnSO_4_ in reducing gene expression of NF-κB p65 and its downstream inflammatory cytokine IL6 by upregulating A20 gene expression via epigenetic modification in the jejunum of progeny. MUC2 is the main intestinal mucin of the mucus layer that protects the integrity of gut epithelial cells and an increase of MUC2 gene expression indicates better gut barrier function. TLR2 is one of the toll-like receptors that plays an important role in the identification of distinct molecular patterns from invading pathogens including bacteria, fungi, parasites, and viruses (Mukherjee et al., 2016). As the intestine is the first line barrier for pathogens, the greater reduction of jejunal TLR2 expression in progeny from MMHAC fed breeders than either ITM or TMAAC fed breeders suggests that maternal MMHAC supplementation probably decreased pathogen challenge in progeny birds. Taken together, these findings suggest that supplementation of the breeder diet with bis-chelated trace minerals, MMHAC, reduces jejunal inflammation and improves the innate immune response and gut barrier function in comparison to ITM and/or mineral amino acid complex.

Another important characteristic in broiler breeder development is bone structure. Bone development occurs early in life. Longitudinal growth in long bones occurs by endochondral ossification and the long bones widen by a process involving intramembranous ossification. At the onset of sexual maturity, osteoblasts undergo a dramatic change, from forming lamellar cortical bone, to producing a woven bone called medullary bone, which is a labile source of calcium for eggshell formation. Medullary bone accumulates rapidly on the surfaces of structural bones, especially leg bones. In early laying stages this is in spicules within the medullary cavities and it continues to accumulate slowly over the remainder laying period (Whitehead, 2004). The medullary bone is weaker than the structural bone, but its bone content is positively correlated with humerus strength (Fleming et al., 1998).

Trace minerals play important roles in bone development. Zinc regulates collagen synthesis (Starcher et al., 1980), osteoblast-associated cellular invasion of cartilage matrix (Dibner et al., 2007) and endochondral ossification (Hatori et al., 1995; Ho et al., 2003; Fraker, 2005). Cu regulates crosslinking of collagen and elastin, which provides tensile strength and elasticity for bone (Carlton and Henderson, 1964). Using organic forms of trace minerals to increase their bioavailability could improve bone mineralization and development. For example, feeding methionine hydroxy analog chelated Zn to breeder hens increased Zn and Ca concentrations in tibias and tibial strength (Min et al., 2019); feeding Zn, Cu, and Mn chelated methionine hydroxy analog to turkeys increased tibial breaking strength and cortical bone width (Ferket et al., 2009). In the current study, overall, most of tibial parameters were not different between groups, indicating that half dose of MMHAC and TMAAC were enough to provide minerals needed for tibial growth and development as compared to high dose of ITM, which is probably also due to the greater bioavailability as shown by increased tibial Zn deposition as reported previously (Richards et al., 2015).

Maternal nutrition can affect embryonic development and progeny growth since all nutrients required for embryo development are acquired by nutrient transfer from the breeders (Richards et al., 2010; Özlü et al., 2018; Emamverdi et al., 2019). More trace minerals could be transferred from breeders to progeny chicks when breeders were fed highly bioavailable source of minerals, MMHAC. This hypothesis is confirmed by a previous study in which Zn-MHAC supplementation to breeders increased Zn deposition in egg yolk and albumin than Zn sulfate (Li et al., 2015). The improvement of progeny growth performance in this study could results from increased amount of trace minerals transferred from breeders to progeny, less inflammation and better gut barrier function and innate immunity. Consistent with this study, supplementation of MHA bis-chelated Zn:Cu:Mn and organic Se to breeder hens increased body weight and FCR of 42-day-old offspring birds compared to the same inclusion levels (50:8:60:0.3 ppm of Zn:Cu:Mn:Se) of ITM from sulfate and Se from Na_2_SeO_3_ (Sun et al., 2012). These findings suggest that supplementation of bis-chelated trace minerals MMHAC in the breeder diet improved progeny growth performance in comparison to ITM and mineral amino acid complex.

In summary, in breeder, supplementation with lower levels of MHA-chelated trace minerals improved egg production performance and egg quality and reduced intestinal inflammation while maintaining tibial development in comparison to ITM and/or mineral amino acid complex. In the resultant progeny, maternal supplementation of bis-chelated trace minerals, MMHAC, in the breeder diet improved growth performance and reduced jejunal inflammation in comparison to ITM and mineral amino acid complex. In conclusion, MHA bis-chelated trace minerals performed better, or similarly, to higher concentrations of inorganic trace mineral and/or similar concentrations of trace mineral amino acid complexes in breeders and progeny in this study.

## Data Availability

The original contributions presented in the study are included in the article/Supplementary Materials, further inquiries can be directed to the corresponding authors.
